# A Multi-Compartment Model for Interneurons in the Dorsal Lateral Geniculate Nucleus

**DOI:** 10.1371/journal.pcbi.1002160

**Published:** 2011-09-29

**Authors:** Geir Halnes, Sigita Augustinaite, Paul Heggelund, Gaute T. Einevoll, Michele Migliore

**Affiliations:** 1 IMT, Norwegian University of Life Sciences, Ås, Norway; 2 Department of Physiology, University of Oslo, Oslo, Norway; 3 Institute of Biophysics, National Research Council, Palermo, Italy; Indiana University, United States of America

## Abstract

GABAergic interneurons (INs) in the dorsal lateral geniculate nucleus (dLGN) shape the information flow from retina to cortex, presumably by controlling the number of visually evoked spikes in geniculate thalamocortical (TC) neurons, and refining their receptive field. The INs exhibit a rich variety of firing patterns: Depolarizing current injections to the soma may induce tonic firing, periodic bursting or an initial burst followed by tonic spiking, sometimes with prominent spike-time adaptation. When released from hyperpolarization, some INs elicit rebound bursts, while others return more passively to the resting potential. A full mechanistic understanding that explains the function of the dLGN on the basis of neuronal morphology, physiology and circuitry is currently lacking. One way to approach such an understanding is by developing a detailed mathematical model of the involved cells and their interactions. Limitations of the previous models for the INs of the dLGN region prevent an accurate representation of the conceptual framework needed to understand the computational properties of this region. We here present a detailed compartmental model of INs using, for the first time, a morphological reconstruction and a set of active dendritic conductances constrained by experimental somatic recordings from INs under several different current-clamp conditions. The model makes a number of experimentally testable predictions about the role of specific mechanisms for the firing properties observed in these neurons. In addition to accounting for the significant features of all experimental traces, it quantitatively reproduces the experimental recordings of the action-potential- firing frequency as a function of injected current. We show how and why relative differences in conductance values, rather than differences in ion channel composition, could account for the distinct differences between the responses observed in two different neurons, suggesting that INs may be individually tuned to optimize network operation under different input conditions.

## Author Summary

The dorsal lateral geniculate nucleus (dLGN) is a part of the visual thalamus. This region contains two types of neurons: thalamocortical neurons and local interneurons. Thalamocortical neurons receive information from the retina and transmit information to visual cortex. The interneurons regulate the activity of thalamocortical neurons through inhibitory connections. This regulation is not properly understood, but it is believed to promote contrast enhancement and other vital visual functions. A powerful tool for development of a mechanistic understanding of dLGN functions is computer models that include the involved neurons, their interconnections and their interactions. Quite sophisticated models are available for thalamocortical neurons, but previous interneuron models are too simple for adequate mechanistic understanding of the functional properties of interneurons. We here present a detailed compartmental interneuron-model based on experimental data. The typical response patterns vary between different interneurons, but also within a given neuron, depending on the stimulus it receives. The model identifies a set of ionic mechanisms that can explain this diversity of activity patterns. In addition to being a useful building block for future network simulations of the dLGN, the model gives useful insight into the operating principles of dLGN interneurons.

## Introduction

The dorsal lateral geniculate nucleus (dLGN) receives input from retinal ganglion cells and transmits processed information to visual cortex. About 75–80% of the neurons in the dLGN are thalamocortical (TC) neurons, also called relay neurons, as they relay information from the retina to the cortex. Local GABAergic interneurons (INs) constitute the remaining 20–25%, and are responsible for most of the intra-nuclear connections [1]. By providing feed-forward inhibition from retinal ganglion cells to TC neurons, INs control the number of visually evoked spikes in TC neurons, and refine the receptive fields of TC neurons (e.g. [2]–[5]). The INs are also important for synchronizing thalamic oscillations [6]–[7].

Comprehensive mathematical network models will likely be important for a comprehensive understanding of the key functional features of early sensory processing [8]–[11]. Due to the relatively high abundance of experimental data, the early visual system has attracted particular interest from theoretical neurobiologists. Several mechanistic network models aimed at mimicking responses of neurons in primary visual cortex, and neurons in the dLGN that provide the feed-forward input to visual cortex, have been developed [12]–[21]. Such network models require (i) detailed information about network connectivity and (ii) mathematical neuron models that capture the salient physiological properties of the individual neurons types.

For dLGN, a host of physiological and anatomical experiments have provided detailed information about the neuronal connectivity, as well as the morphology and physiology of TC neurons and INs (see *e.g*. reviews in [22]–[24]). Different electrophysiological characteristic of TC neurons have been captured in a series of modeling works [25]–[31], and the accumulated insight has been incorporated in a high-resolution model which comprises a detailed description of the cell morphology, a set of different ion channels and their distribution over the somatodendritic membrane [32]. For the INs, the situation is more problematic: models are few and less detailed [33]–[36]. Until now, network models for the early visual system have either omitted INs entirely, or represented them in a very simplified manner (but see [37]). A satisfactory theoretical understanding of the computational properties of the dLGN circuit, and thus also the input to visual cortex, will likely require network models incorporating more detailed IN models. The development of such models is the topic of the present paper.

INs may exhibit a rich variety of firing patterns, including (i) initial “sags” by hyperpolarizing current injections, (ii) rebound bursts when released from hyperpolarization, (iii) tonic firing of action potentials (APs) by depolarizing current injections, (iv) initial bursts by depolarizing current injections, (v) spike-time adaptation during depolarizing current injections, and (vi) periodic bursting during depolarizing current injections [36], [38]–[44]. Most previous models focus on aspects of passive signal propagation in INs [33]–[35]. To our knowledge, the only currently available model that includes a variety of the active mechanisms in INs was primarily developed in order to study the mechanisms behind the rebound bursts [36], whereas other properties such as dendritic conductances and the relationship between somatic current injections and action potentials frequency (hereby referred to as the I/O curve) were not taken into account.

The morphology and distribution of dendritic ion channels are crucial for the integration of synaptic input (reviewed in [45]), and can even influence the neuron's response to somatic current injections [46]–[49]. Several active conductances have been identified in the dendrites of INs [38], [50]–[54]. Dendritic ion channels are likely of particular importance in INs, as their dendrites have not only postsynaptic contacts for excitatory retinal and cortical input, but also presynaptic terminals for inhibitory output to TC dendrites [2]. An understanding of how INs provides feed forward inhibition to TCs thus requires models that incorporate the electrically active processes in the dendritic tree. No previous IN model includes such properties.

We here propose a new and detailed multi-compartment model of the IN, which advances previous models in several aspects. Firstly, it includes a detailed 3D reconstruction of IN morphology, and a set of somatic and dendritic active conductances which could reproduce some of the key response patterns of INs (including (i)-(vi) listed above). In this way it lays the foundation for simulating active dendritic signaling on a fine spatiotemporal scale. Secondly, we present two different parameterizations (P1 and P2) of the model, which were constrained by current-clamp data from two example neurons (IN1 and IN2). This approach allowed us to do a comparative study, and relate differences in the parameterizations P1 and P2 to differences in the firing patterns between IN1 and IN2. The two parameterizations contain the same set of ion channels such that differences in the responses of the two neurons could be explained solely in terms of relative differences in the peak values of the conductances. Thereby, we demonstrate that relative differences in conductance values of the included ion channels may account for the substantial variations in firing patterns observed between INs.

Finally, we were able to explain the experimentally observed responses in different neurons (IN1 or IN2) under 8 different input conditions, obtaining quantitative agreement with the experimental I/O-curves over the entire input range studied. This is a significant advance compared to previous models of similar cells. Since the overall input to any given cell in a network generally varies with time over a wide dynamic range, our model will likely allow a more accurate representation of the integration and computational properties of these neurons when included in a realistic dLGN network.

Preliminary results from this model have previously been presented in abstract form [55]. The model will be publicly available on ModelDB (http://senselab.med.yale.edu/modeldb).

## Methods

### Experimental procedures

Brain slices containing dLGN were prepared from GAD67-GFP (Δneo) knock-in mice [56] in accordance with the guidelines and approval of the Animal Care Committee in Norway. Mice, 29–33 days old, were deeply anaesthetized with halothane and sacrificed by rapid decapitation. A block of the brain was dissected out and 250–300 µm thick coronal slices were cut in 4°C oxygenated (5% CO_2_–95% O_2_) solution containing (mM): 75 glycerol, 87 NaCl, 25 NaHCO_3_, 2.5 KCl, 0.5 CaCl_2_, 1.25 NaH_2_PO_4_, 7 MgCl_2_, and 16 D-glucose, and kept submerged in oxygenated (5% CO_2_–95% O_2_) artificial cerebrospinal fluid (ACSF) containing (mM): 125 NaCl, 25 NaHCO_3_, 2.5 KCl, 2 CaCl_2_, 1.25 NaH_2_PO_4_, 1 MgCl_2_ and10 D-glucose at 34°C for at least 30 min before the experiment. During experiments, slices were kept submerged in a small (∼1.5 ml) chamber and perfused with ACSF at the rate of 5 ml min^−1^ heated to at 36°C through an inline heater. In some of the experiments, as indicated, 4-Ethylphenylamino-1,2-dimethyl-6-methylaminopyrimidinium chloride (ZD 7288; 20 µM; Tocris Bioscience, Bristol, UK) was included in the perfusion ACSF to block the hyperpolarization-activated cation current *I*_h_.

Whole-cell voltage- or current-clamp recordings were made from INs in dLGN. Neurons were visualized using DIC optics and infrared video microscopy. INs were identified by expression of GFP which was specifically expressed in GABAergic neurons under control of the endogenous GAD67 promoter in the GAD67-GFP knock-in mice [56] we used. Recordings were obtained with borosilicate glass electrodes (4–6 MΩ) filled with (mM): 115 potassium gluconate, 20 KCl, 10 HEPES, 2 MgCl_2_, 2 MgATP, 2 Na_2_ATP, 0.3 GTP (pH adjusted to 7.3 with KOH). For morphology reconstruction, biocytin (0.25%; Sigma-Aldrich, St Louis, USA) was included in the intracellular solution.

Current traces were recorded and filtered at 3 kHz with a HEKA EPC 9 amplifier (HEKA Electronik, Lambrecht, Germany), while voltage traces were recorded and filtered at 10 kHz with an Axoclamp 2A amplifier (Molecular Devices, Palo Alto, CA, USA).

After recordings, slices were fixed by 0.1 M phosphate buffer containing 4% paraformaldehyde and kept there for at least 24 h. After biocytin histochemistry with avidin-biotin complex (Vectastain ABC kit, Vector Laboratories, Inc, USA) and diaminobenzidine (DAB; Sigma-Aldrich, St. Louis, USA), interneurons were drawn under a x100 objective using software for neuron reconstruction (Neurolucida, MicroBrightField, Inc. USA).

### Computer modeling

#### Morphology

**Figure 1:**
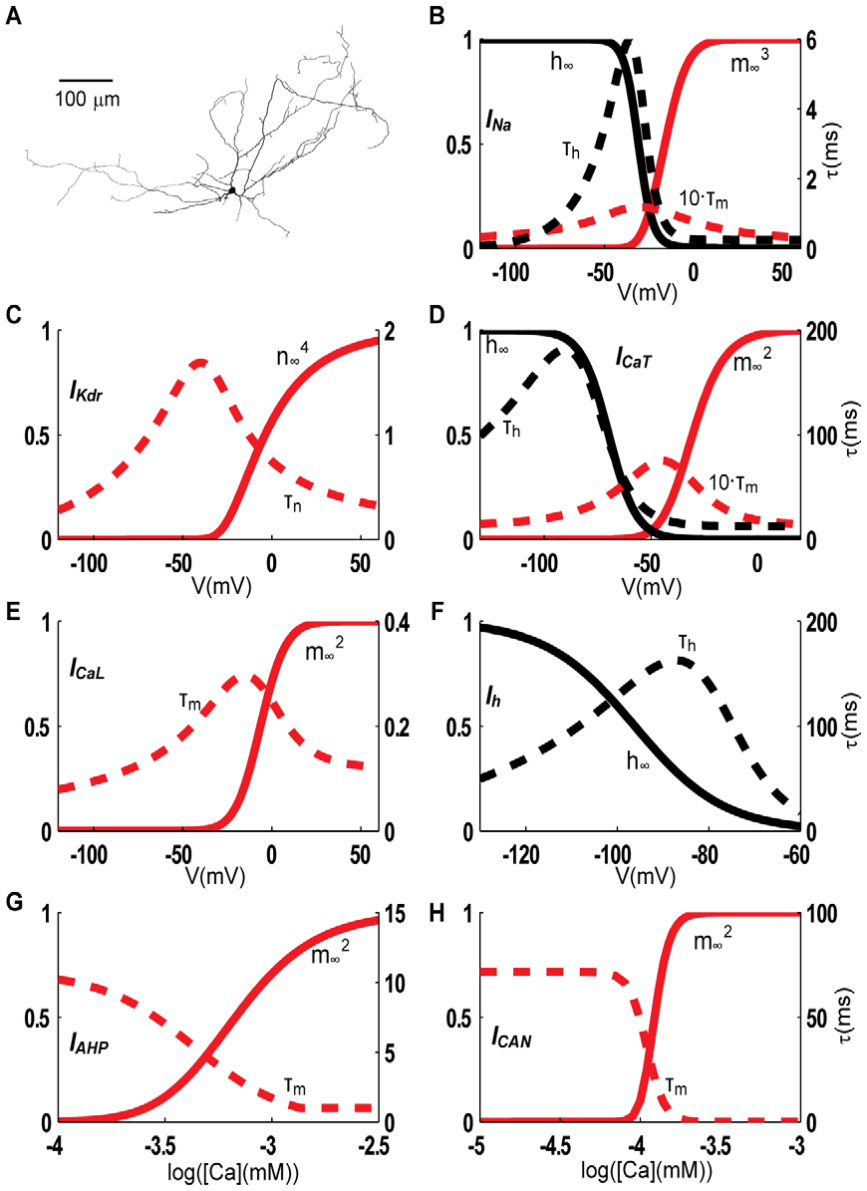
Morphology and ion channel kinetics. The same dLGN morphology (**A**) was used in all simulations. The steady state values of activation/inactivation variables (red/black full lines), along with the activation/inactivation time constants (red/black dotted lines) are plotted as a function of voltage for voltage dependent ion channels (**B–F**), and as a function of intracellular calcium concentration for calcium dependent ion channels (**G–H**). *Na* and *K_dr_* kinetics are shown for the parameterization P1 of the model. With respect to this, P2 kinetics was shifted +2.3 mV and +3.2 mV for *Na* and *K_dr_* respectively. For all other ion channels, the same kinetics applies to both parameterizations (P1 and P2).

In this study, we use our own 3D reconstructions of the morphology of mouse INs. The model was based on the selected morphology shown in Figure 1A, which is used in all simulations unless otherwise stated. The total surface area was 9864 µm^2^, the total length of dendrites was 5771 µm, the longest dendrite was 673 µm, and the mean somatodendritic diameter was about 0.5 µm. The model morphology contained 104 sections that were split into a total of 330 segments. Additional test simulations were run using morphologies of (a) a similar (9566 µm^2^), (b) a smaller (7071 µm^2^), and (c) a larger (14336 µm^2^) total membrane area compared to the original morphology (o). Axons in LGN INs are generally very thin, and could not be identified in the morphology data. Given the small surface area of the axon, it is not expected to affect somatic input/output-data significantly. All simulations were carried out using the *NEURON* simulation environment [57]. The model files are available for public download from the ModelDB section of the Senselab database (http://senselab.med.yale.edu).

#### Passive properties

The axial (cytoplastic) resistivity (*R_a_*) was taken from the literature, *R_a_* = 113 Ωcm [35], which also was close to the value used in earlier models of (passive) dLGN dendrites (*R_a_* = 100 Ωcm in [33], [34], [36]). The reason for using a fixed value for *R_a_* is that this parameter is not well constrained by somatic voltage recordings, as the response has a low sensitivity to this parameter [34], [58]. The remaining passive properties, the membrane capacitance (*C_m_*), the membrane resistance (*R_m_*), and a leakage current specified by its conductance (*g_pas_ = 1/R_m_*) and its reversal potential (*E_pas_*) were estimated for each neuron by a manual trial and error process, to obtain a qualitative fit of all responses to hyperpolarizing or small depolarizing current injections in the soma. The fit procedure also included the hyperpolarization- activated cation current (*I_h_*) and a low-threshold calcium channel (*Ca_T_*), which are active around rest and can influence the input resistance (*R_IN_*) and membrane time constant (*τ_m_*) of the neuron [35]. We found that the two neurons (IN1 & IN2) had different passive properties. The final set of parameters are listed in Table 1, and are within the physiologically relevant ranges suggested earlier [59]. Note that *E_pas_* is different from the resting potential (*V_rest_*), due to active conductances that are nonzero around rest.

**Table 1:** Parameter sets (P1 and P2) adapted to the response patterns of two INs (IN1 and IN2).

Parameter	Description	*g_dend_/g_soma_* * *^)^*	P1	P2
*V_rest_*	Resting potential		-63 mV	-69 mV
*R_m_*	Membrane resistance		22 kΩcm^2^	45 kΩcm^2^
*C_m_*	Membrane capacitance		1.1 µF/cm^2^	1.1 µF/cm^2^
*R_a_*	Axial resistivity		113 Ωcm	113 Ωcm
*R_IN_*	Total input resistance		240 MΩ	470 MΩ
*τ_m_*	Membrane time constant		24 ms	50 ms
*g_Na_*	Max. *Na*-conductance in soma	0.1	0.09 S/cm^2^	0.09 S/cm^2^
*Sh_Na_* ** *^)^*	Shifts in *Na*-kinetics		+10.4 mV	+12.7 mV
*g_Kdr_*	Max. *K_dr_* -conductance in soma	0.1	0.37 S/cm^2^	0.5 S/cm^2^
*Sh_Kdr_* ** *^)^*	Shifts in *K_dr_* –kinetics		+11.8 mV	+15 mV
*g_CaT_*	Max. *Ca_T_*-conductance in soma	1+0.04*·x*	1.2·10^−5^ S/cm^2^	8.5·10^−6^ S/cm^2^
*g_CaL_*	Max. *Ca_L_*-conductance in soma	0.25	9·10^−4^ S/cm^2^	1.3·10^−3^ S/cm^2^
*g_h_*	Max. *I_h_*-conductance in soma	1	1.1·10^−4^ S/cm^2^	1·10^−5^ S/cm^2^
*g_AHP_*	Max. *I_AHP_*-conductance in soma	0.1	6.3·10^−5^ S/cm^2^	1.3·10^−4^ S/cm^2^
*g_CAN_*	Max. *I_CAN_* -conductance in soma	1+0.04·*x*	2·10^−8^ S/cm^2^	1·10^−7^ S/cm^2^

*) Ion-channel distributions are described as the ratio between dendritic and somatic maximum conductance values. Ion channel density was assumed to be uniform over the dendrites, except for *Ca_T_* and *I_CAN_*, where the densities were assumed to increase linearly with distance (*x* [µm]) from the soma.

**) Shifts are given in the positive direction along the voltage axis, and relative to the kinetics curves in Traub et al. 1993.

#### Ion channel kinetics

**Figure 2:**
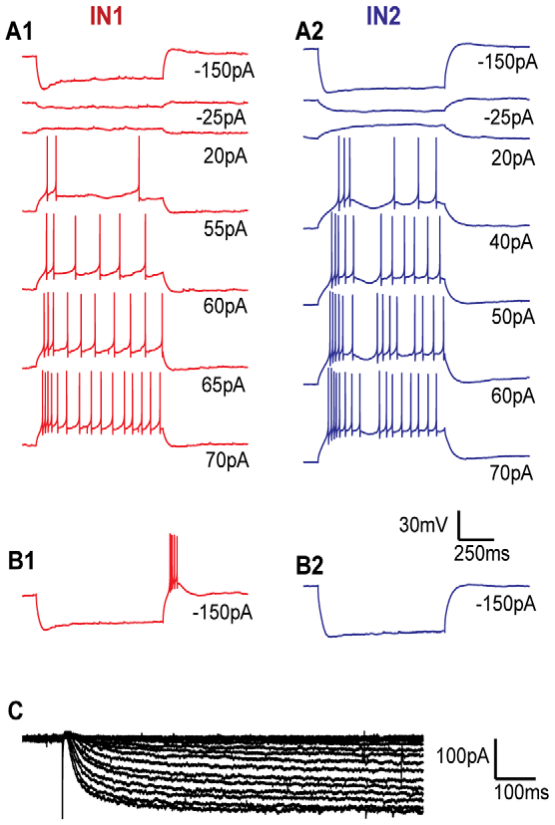
Experimental data. Somatic voltage responses in IN1, resting at -63 mV (**A1**), and IN2, resting at -69 mV (**A2**) to 7 current pulses of different intensity. When IN1 was held at -57 mV, a strong hyperpolarizing current injection was followed by a rebound burst (**B1**). When IN2 was held at -58 mV, a strong hyperpolarizing current injection did not cause a burst (**B2**). Current injections were applied as 900 ms step pulses to the soma, with intensities as indicated below the traces. Two repetitions were made of each CC-experiment, whereof one is shown. The voltage scale bar applies to all panels (A-B). *I_h_* was measured at different potentials from -130 mV with 5 mV steps, in three different neurons. Recordings are shown for one neuron (**C**).

Seven different active ion channels were included in our model, the selection of which was based on previous literature and our own simulations (see Results). The ion channels include the traditional Hodgkin-Huxley sodium- and delayed-rectifier potassium channels (*Na* and *K_dr_*), a hyperpolarization- activated cation channel (*I_h_*), a low-threshold, T-type calcium channel (*Ca_T_*), a high-threshold, L-type calcium channel (*Ca_L_*), a medium-duration, calcium-dependent afterhyperpolarization channel (*I_AHP_*), and a long-lasting calcium-activated non-specific cation channel (*I_CAN_*). Ion channel kinetics is summarized in Figure 1B-H. Ion channels were inserted in the somatic and dendritic membrane, and modeled at a temperature of 36°C. Standard Hodgkin-Huxley formulation was used [60], but with the Goldman-Hodgkin-Katz formulation [61] for calcium channels instead of a reversal potential-based current. The included ion channels are described below. Experiments have characterized a slow hyperpolarization-activated cation conductance (*I_h_*) in rat INs [41]. The kinetics of *I_h_* in that study was too slow to account for observations in our current-clamp data (Figure 2). We therefore performed voltage clamp experiments to measure *I_h_*, and used these measurements to determine the activation kinetics (see Results). We assumed a reversal potential of -44 mV, as was earlier found in rat INs [41]. APs were generated using standard Hodgkin-Huxley-type sodium (*Na*) and potassium (*K_dr_*) channels. The channels we used were originally adapted for hippocampal neurons [62], but have since then been used successfully in modeling a variety of cell types [63]. In order to be consistent with the threshold for AP initiation in the data sets, the voltage dependence of activation and inactivation was shifted compared to the original values [62]. As IN1 and IN2 had different firing thresholds, the *Na* kinetics was shifted +10.4 mV (i.e. in the positive direction along the voltage axis) in P1, and +12.7 mV in P2, while the *K_dr_* kinetics was shifted +11.8 mV in P1 and +15 mV in P2. The kinetics for P1 is shown in Figure 1B-C. The low-threshold, T-type calcium channel (*Ca_T_*) needs hyperpolarization to lift the inactivation. Therefore, it is most active shortly after hyperpolarization, and then generates a calcium-dependent depolarization envelope upon which a (rebound) burst of APs may ride [36], [38], [43], [44], [64]. *Ca_T_* kinetics was adapted to recent voltage clamp data for INs [44], and corrected for temperature differences (24–36°C) using Q10 values of 3.0 and 1.5 respectively, for activation and inactivation [36]. Activation kinetics had to be shifted +8 mV in order to prevent excessive *Ca_T_* active at rest, and in order to agree with the observed bursting behavior in both example neurons (IN1 and IN2). A high-threshold, L-type calcium channel (*Ca_L_*) opens mainly during APs. *Ca_L_* regulates tonic firing, mainly by increasing intracellular calcium levels that trigger calcium-dependent potassium currents such as *I_AHP_* [36], but may also be important for dendritic signal propagation, and for triggering dendritic GABA-release [53]. *Ca_L_* kinetics was adapted from [36], with activation kinetics shifted +7 mV in order to avoid substantial *Ca_L_* activation at potentials below the AP-firing threshold. A voltage- insensitive medium-fast afterhyperpolarization current (*I_AHP_*) was used to modify spike frequencies for depolarizing current injections. Medium-fast *I_AHP_* (reviewed in [65], [66]) is calcium dependent, reaches half activation in the 400–800 nM range, has a relatively fast time to peak (1–5 ms), and decays with a time-course dependent on the amount of calcium influx [65], [66]. The kinetics for *I_AHP_* was modified from [67], so that *I_AHP_* reaches half activation in the appropriate range ([*Ca*]*_i,half_* = 435 nM) with a time constant of about 3 ms (Figure 1G). A long-lasting calcium-activated nonspecific cation current (*I_CAN_*) is known to provide afterdepolarization in other thalamic neurons [68], [69]. *I_CAN_* is present in INs [36], [52], where it may be involved in prolonged bursts [36]. The kinetics for *I_CAN_* was taken from [36].

#### Ion channel distribution

Quantitative data on the sub-cellular ion channel distribution in INs is only available for *Ca_T_* and *Ca_L_* [43], [50], [51]. For the remaining ion channels we assumed a uniform distribution over the dendrite (with one exception), generally with a different density from that in the soma. The ratios between dendritic- and somatic ion channel density (*g_dend_/g_soma_*) were based on relevant literature, as specified below. Experiments suggest that *I_h_* channels play an important role in dendrites of INs [52]. We make the same assumption as in a previous model for TC neurons [32], that *I_h_* channels are uniformly distributed over the soma and dendrites (*g_dend_/g_soma_ = 1*). Recent experiments have shown that dendritic AP propagation in INs is supported by *Na* and *K_dr_* channels [53], [70]. However, increases in the rise time and duration of the AP waveform during propagation, indicate that dendritic densities may be lower than those in the soma [70], which is also the case in TC neurons [71]. We assumed that dendritic *Na*- and *K_dr_* densities were 10% of those in the soma (*g_dend_/g_soma_ = 0.1*). The higher somatic densities may also reflect contributions from the initial axonal segment which was not modeled explicitly. In line with experimental data, we assumed that the density of dendritic *Ca_L_*-channels was 25% of that in the soma, and that the dendritic density did not decrease with distance from soma [51]. *Ca_T_*-channels in the dLGN are preferentially expressed in the dendritic membrane [43], [50]. Their distribution may be important for determining the threshold value of the low-threshold calcium spike [48], [72]. According to experiments, the density of *Ca_T_*-channels increases linearly with distance from the soma, and reaches 239% of the somatic value at 60 µm [50], which is approximately 4%/µm. We therefore assumed a distance-dependent *Ca_T_* -density, given by *g_dend_(x)  =  g_soma_·*(*1+0.04·x*), where *x* [µm] denotes the distance from soma. Medium-fast *I_AHP_* channels are mainly influenced by calcium entering through high-voltage-activated calcium channels [66], and due to their relatively short time to peak, it has been argued that *I_AHP_* channels are located close to the point of calcium influx [65]. In Hippocampal neurons, *I_AHP_* channels are selectively coupled to *Ca_L_* channels [73], as has also been suggested for INs [36]. We obtained such a functional coupling by assuming that *I_AHP_* and *Ca_L_* both have their peak density in the somatic region, using the distribution *g_dend_/g_soma_ = 0.1* for *I_AHP_*. As *Ca_T_* channels are preferentially expressed in the dendrites, they will have a smaller impact on *I_AHP_*. *I_CAN_* conductances were not apparent in dissociated cells, suggesting a dendritic location for these ion channels [36]. Studies have suggested that *I_CAN_* is mainly activated by calcium entering through *Ca_T_* channels [36], [43], [74]. This may be explained by the two channel types having similar spatial distribution. As for *Ca_T_*, we thus assumed an *I_CAN_* -distribution given by *g_dend_(x) = g_soma_·*(*1+0.04·x*), where *x* [µm] denotes the distance from soma.

#### Intracellular calcium dynamics

The intracellular calcium level (relevant for the gating of *I_AHP_* and *I_CAN_*), was modeled as a leaky integrator [27], [75], [76]:
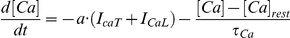



The factor *α* converts the calcium currents *I_CaT_* and *I_CaL_* to a calcium-concentration increase in a small volume immediately inside the membrane. We specified *α* = 0.0155 mmol/(cm·C) in all compartments, which gave rise to calcium levels between 50 nM and 100 nM around resting potential (depending on resting potential and *Ca_T_* conductance), and up to about 2 µM during rapid spiking/bursts, which is within the concentration ranges obtained in similar treatments (e.g. [28], [76], [77]). The various extrusion and buffering mechanisms are described collectively by a first-order decay process. We chose a basal calcium concentration [*Ca*]*_rest_* = 50 nM and a decay rate *τ_Ca_* = 50 ms, which give rise to similar calcium decay as in the previous IN model [36].

#### Synaptic input

About 1/4 of the total synaptic input to INs is GABAergic, most likely from other INs and from interneurons in the perigeniculate nucleus and the thalamic reticulate nucleus [78], [79]. The GABAergic responses in INs are predominantly mediated by GABA_A_-receptors [40], [79]. GABAegic terminals on INs form equal numbers of synapses on dendrites and dendritic appendages, and somewhat fewer in the soma region [78]. We therefore implemented a physiologically plausible hyperpolarizing input through GABA_A_-receptors that were randomly distributed in the dendritic tree. The synapses were modeled as a sum of exponentials with rise and decay times of 0.5 ms and 5 ms, respectively, based on values found for GABA_A_-receptors in other neurons [67], [80], [81]. For the reversal potential, we used -82 mV, which has been measured for the GABA_A_-response in INs [79].

## Results

### Empirical data for constraining the model

The set of empirical data used for constraining the model is presented in Figure 2. The firing patterns in two different INs (IN1 and IN2) under 8 different experimental conditions are shown in Figure 2A–B.

IN1 had a resting potential of -63 mV (Figure 2A1). The onset of a strong hyperpolarizing current injection (-150 pA) made the membrane potential drop rapidly to a hyperpolarized peak value, before it increased to a less hyperpolarized plateau value. This initial sag is a trademark of the *I_h_* current. IN2 had a resting potential of -69 mV (Figure 2A2). The initial sag for hyperpolarizing current injections was less pronounced in IN2 than in IN1.

Depolarizing stimuli gave rise to an initial, transient response (characterized by a high AP-firing frequency), followed by regular AP- firing at lower frequency. In IN2 the initial response tended to be distinct and burst-like (see e.g. Figure 2A2, 40 pA), whereas IN1 tended to have a more gradual transition between the initial response and the regular AP-firing (see e.g. Figure 2A1, 70 pA). We did not quantify these differences, but use the term *initial burst* for all initial responses that can be distinguished from the later, more regular AP firing. IN1 needed stronger depolarizing input (≥55 pA) to initiate AP-firing than IN2 (≥40 pA). However, IN1 had the steepest I/O-curve (see below), so that both neurons had about the same firing frequencies when stimulated at 70pA.

In one experiment, small current injections were used to hold the membrane potential at a depolarized value (-57 mV in IN1 and -58 mV in IN2). In this case, strong hyperpolarizing current pulses (-150 pA) were followed by a rebound burst in IN1 (Figure 2B1), but not in IN2 (Figure 2B2).

### Voltage dependence of *I_h_*-activation

The *I_h_* -current in rat INs has no calcium dependence [82] and has been measured and thoroughly described [41]. A comparison of the time course of the initial sag for recordings from rat INs [36], [41] with ours from mouse INs, especially IN1 (Figure 2A1), indicated that *I_h_* may differ between the two species. We therefore recorded the *I_h_* response to different hyperpolarizing voltage steps in our mouse INs (Figure 2C), and used these data to estimate the *I_h_* kinetics.

**Figure 3:**
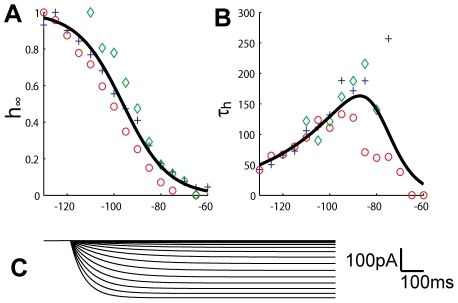
*I_h_*-kinetics. The steady state values of *I_h_*-activation (**A**), and activation time constant (**B**), fitted to data from three INs (plusses, circles and diamonds). A simulation of the *I_h_*-current at different step potentials between -130 and -65 mV, with 5mV steps (**C**) gives similar traces to those seen experimentally (see Figure 2C).

We derived *I_h_* -activation curves for mouse INs using the following procedure [41]: With *I_max_* denoting the maximum observed amplitude throughout the trial (e.g., about -180 pA in the dataset shown in Figure 2C), and *I_inf_* denoting the steady-state value of the response for a given command potential (*V)*, the ratio *I_inf_/I_max_* was plotted against *V* for three data sets (Figure 3A). The data points were then fitted by a Boltzmann curve (Figure 3A; full line), given by: *I_inf_/I_max_ = (1+exp((V-shift)/stp))^−1^*, where s*hift* is the potential at half-inactivation, and *stp* determines the steepness of the activation curve. Using the inbuilt optimizer *fminsearch* in Matlab, we obtained the optimal parameters *shift = -96 *mV and *stp = 10 *mV. The corresponding values estimated for rat INs were -79 mV and 7.4 mV [36], [41].

**Figure 4:**
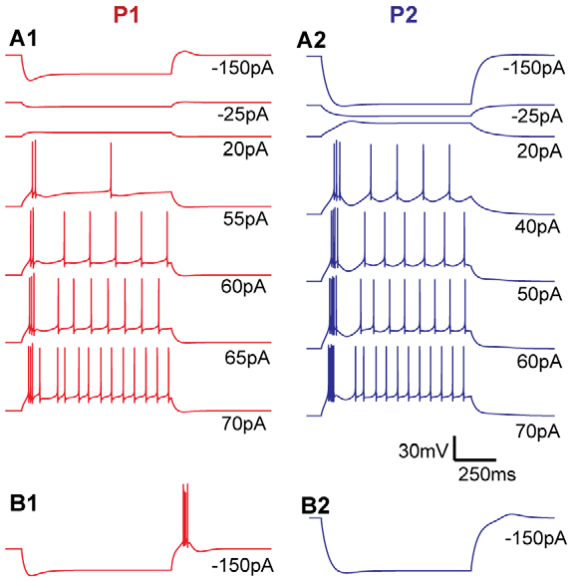
Simulation results. Somatic voltage responses in P1, resting at -63 mV (**A1**), P2, resting at -69 mV (**A2**), P1, starting from -57 mV (**B1**), and P2, starting from -58 mV (**B2**) reproduce the essential features of the experimental recordings from IN1 and IN2 (Compare with Figure 2). The voltage scale bar applies to all panels.

In order to determine the voltage dependence of the time constants for steady-state activation, the current traces (interval from 0–1000 ms after stimulus onset in Figure 2C) were fitted by exponential curves on the form *I_h_ = (1-exp(-t/τ_h_))·I_inf_*. Only a single exponential term was used, as it yielded a good fit. In this way we estimated the time constant (*τ_h_*) at each command potential as plotted in Figure 3B. The data points for *τ_h_* were in turn fitted with a bell shaped curve (as in [41]) of the form: *τ_h_(V) = exp[(V+a1)/a2]/(1+exp[(V+a3)/a4])*, with *V* measured in mV and *τ_h_* in ms. Using *fminsearch*, we obtained the optimal fit for [*a1, a2, a3, a4*] = [250, 30.7, 78.8, 5.78]. Note that the maximum value of *τ_h_* is about 200 ms, while the corresponding value found for rat INs was about 1000 ms [36], [41]. The faster kinetics was in good agreement with the time course of the initial sag (see Figure 4).

### Models of two INs

We sought two parameterizations of the model (P1 & P2), which separately should explain the characteristic features of the respective data sets (IN1 and IN2) under all 8 experimental conditions (Figure 2A–B). As an additional constraint we assumed that the two neurons contained the same types of ion channels and had the same axial resistivity (*R_a_*). This means that we aimed to explain the differences between IN1 and IN2 in terms of differences in three passive parameters (*R_m_*, *E_pas_, C_m_*), seven parameters representing the density of ion channels (*g_h_, g_Na_, g_Kdr_, g_CaT_, g_CaL_, g_AHP_, g_CAN_*), and two parameters (*Sh_Na_*, *Sh_Kdr_* ) representing shifts in the activation/inactivation curves of *Na* and *K_dr_* along the voltage axis. Shifts in the kinetics of the AP-generating currents were allowed to be free parameters as we observed clear differences in spiking threshold between neurons IN1 and IN2. In order to limit the number of free parameters, the voltage and calcium dependence of the remaining ion channels were assumed to be identical in the two neurons.

**Figure 5:**
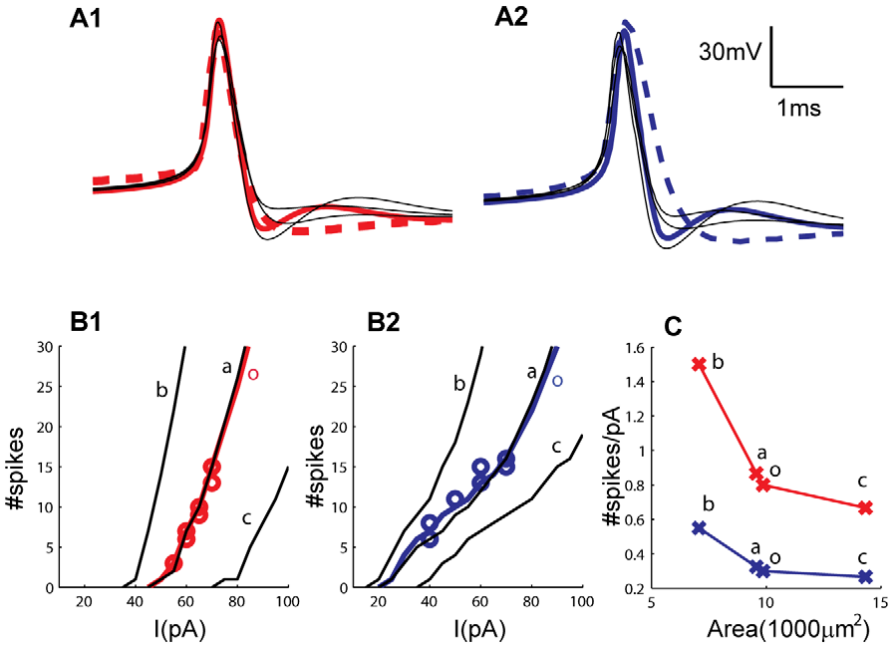
AP waveforms and I/O curves. Experimental (dashed colored lines) and simulated (thick, colored lines) AP waveforms for IN1/P1 (**A1**) and IN2/P2 (**A2**). The morphology did not significantly affect the simulated AP-waveform (simulations with three alternative morphologies are shown by thin, black lines). The simulated (thick, colored lines) and experimentally obtained (circles, two repetitions for each stimuli) I/O curves for P1/IN1 (**B1**) and P2/IN2 (**B2**) were in agreement. Simulations are also shown for alternative morphologies (thin, black lines), with (a) similar, (b) smaller and (c) larger membrane area compared to the original morphology (o). The slopes of the I/O curves decreased with membrane area, but the essential differences between P1 and P2 were preserved (**C**). I/O curves were defined as #spikes elicited throughout the stimulus period as a function of the amplitude of the injected current. Slopes of I/O curves were always calculated in the range between 2 and 15 elicited APs.

The experimental protocols for the two example neurons (Figure 2A–B) were replicated in the simulations shown in Figure 4, where we show that many significant features of the experimental results are reproduced by the model using the two sets of parameters (P1 and P2, Table 1). P1 had resting potential -63 mV, and P2 had resting potential -69 mV (as in the empirical data sets). Stimulus intensities between -150 pA and 20 pA gave rise to sub-threshold responses in both models. The higher response amplitudes in P2 were due to the significantly higher membrane resistance found for this neuron. Initial sags in P1 and P2 for strong hyperpolarizing current injections (-150 pA) were due to *I_h_*. Simulated action potentials had a width (at half max response) of about 0.4 ms in P1 as well as in P2 (Figure 5A), which is typical for INs [39], [40], and agreed well with the AP width of IN1. The APs elicited by IN2 were somewhat broader, and had a width of about 0.6 ms. We did not change the kinetics of *Na* and *K_DR_* channels to account for the variability in AP shapes. The mechanisms behind other characteristics in the response patterns are discussed in the following subsections.

### I/O-curves

As in the data sets (Figure 2A–B), depolarizing current injections to the model (55–70 pA to P1 and 40–70 pA to P2) gave rise to an initial burst (or a few APs with short intra-spike intervals) followed by regular activity with a lower AP firing frequency (Figure 4A).

The slope of the I/O curve was mainly regulated by the interplay between calcium entering through *Ca_L_* channels and a single, calcium activated potassium channel (*I_AHP_*). As the high-voltage activated *Ca_L_* channels open during APs, the intracellular calcium concentration will accumulate during high firing frequencies, so that also *I_AHP_* increases with firing frequency. In this way the *Ca_L_*/*I_AHP_*-mechanism flattens the I/O curves, as has been well described in earlier modeling studies (e.g. [83]). Without this regulatory mechanism, the firing frequency (as resulting from the *Na* and *K_dr_* channels) was generally too high, and the I/O curves were too steep compared to the data in Figure 2A (results not shown).

**Figure 6:**
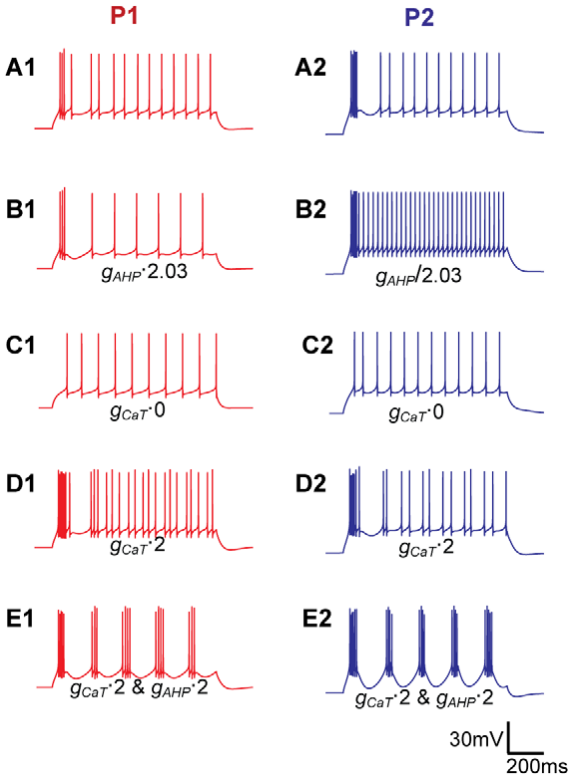
Mechanisms behind initial bursts and regular spiking. All panels show responses to a 70 pA depolarizing current injections. As a reference, the responses in (**A**) use the original parameters for P1 and P2, and are the same as in Figure 4. In P2, the initial burst and the regular spiking are separated by a pronounced afterhyperpolarization (**A1**), while in P1 the transition between the initial burst and the regular spiking is more gradual (**A2**). These characteristics were interchanged between P1 and P2 when *g_AHP_* was interchanged (i.e., multiplied/divided by a factor 2.03 in P1/P2) between the two parameterizations (**B**). Initial bursts were eliminated when *g_CaT_* was set to zero (**C**), and became stronger when *g_CaT_* was increased by a factor 2 (**D**). Increasing *g_AHP_* and *g_CaT_* by a factor 2 gave rise to periodic bursting in both neurons (**E**). The scale bar applies to all panels. When conductance values were changed, the resting potential was kept at the original level by small compensatory current injections.

With parameters as in Table 1, the I/O curves of P1 and P2 agreed well with the data sets, being steeper in P1, as the conductance values for both these channels (*g_CaL_* and *g_AHP_*) were higher in P2 (Figure 5B). In order to investigate the impact of *I_AHP_*, we interchanged the conductance values (*g_AHP_*) between P1 and P2, leaving all other parameters at their original values. The high *g_AHP_* resulted in a lower spike frequency in P1 (Figure 6B1), and in a threefold reduction in the slope of the I/O-curve (from the original ∼0.8 spikes/pA to ∼0.25 spikes/pA (results not shown)). Correspondingly, the low *g_AHP_* increased the spiking frequency in P2 (Figure 6B2), and made the I/O curve three times steeper (from the original ∼0.3 spikes/pA to ∼0.9 spikes/pA).

### Initial bursts

The initial bursts for depolarizing current injections were well reproduced by the model (Figure 4A), and depended mainly on *Ca_T_*. The *I_h_* conductance had negligible impact on these initial bursts (results not shown), but the bursts vanished when *g_CaT_* was set to zero (Figure 5C), and became stronger when *g_CaT_* was increased (Figure 5D). Despite P1 having the highest *g_CaT_*, the initial bursts were most pronounced in P2. This may seem counterintuitive, but we found that this is mainly due to differences in resting membrane potential. The lower resting potential in P2 means less inactivation of *Ca_T_* at rest, and compensates for the lower *g_CaT_*. Note that our *Ca_T_* kinetics (Figure 1D) was shifted +8 mV compared to the empirical data set for rats [44]. Using the *Ca_T_* activation from that empirical study would thus give even stronger bursts (Figure 2A).

### Transition between initial bursts and regular firing

Due to the high intra-burst firing frequencies, initial bursts gave rise to strong *I_AHP_* activation, resulting in a period of afterhyperpolarization which distinctly separated initial bursts from the regular AP firing. The afterhyperpolarization was most pronounced in P2, as P2 had the higher *g_AHP_* (Figure 4A), but became most pronounced in P1 when *g_AHP_* was interchanged between the two parameterizations (Figure 6B).

Previous experiments have shown that some INs respond to depolarizing input by periodic bursting [42], [52]. Although periodic bursting was not observed in our experiments (IN1 and IN2), we ran test simulations to see if the mechanism that explained the initial bursts and subsequent afterhyperpolarization in IN1 and IN2 also could give rise to periodic bursting. Using the parameter sets P1 and P2 as a starting point, an increase in *g_CaT_* (by a factor 2) increased the intensity of the initial burst, but also the overall AP firing frequency, suggesting a nonzero *Ca_T_*-activity throughout the stimulus period. In P2, the increased *g_CaT_* also gave rise to a periodic firing of pairs of APs (Figure 6D2), indicating a periodic interplay between *Ca_T_*-driven bursts and *I_AHP_*-driven afterhyperpolarizations. By also increasing *g_AHP_* (by a factor 2), both the afterhyperpolarization (triggering the bursts) and the bursts (triggering the afterhyperpolarization) became more intense, and periodic bursting was obtained in both P1 and P2 (Figure 6E). The model thus predicted that the interplay between *Ca_T_* and *I_AHP_* could explain the periodic bursting observed in some INs [42], [52], and that periodically bursting neurons have high *Ca_T_* and *I_AHP_* conductances relative to neurons that do not show this behavior (e.g. IN1 and IN2).

### Rebound bursting during physiological conditions

Small current injections (22 pA in P1 and 12 pA in P2) were used to shift the membrane potential from rest to the holding potentials of -57 mV in P1, and -58 mV in P2. When held at these depolarized potentials, strong hyperpolarizing current injections (-150 pA) were followed by a rebound burst in P1, but not in P2 (Figure 4B).

**Figure 7:**
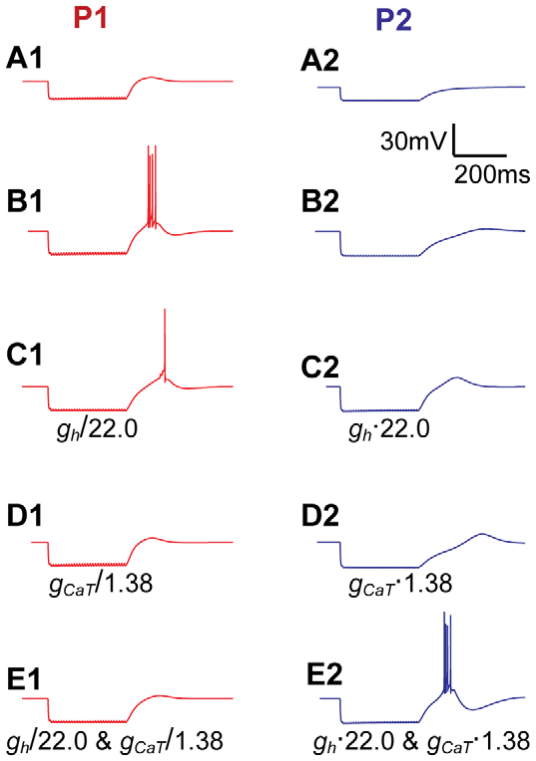
Mechanisms behind rebound bursting. All panels show responses to strong hyperpolarizing synaptic input (50 synapses, activated with 10 ms intervals, during 300 ms). Rebound bursts were not elicited by P1 (**A1**) or P2 (**A2**), starting from their normal resting potentials of -63 mV and -69 mV, respectively. From a -57 mV holding potential, P1 (**B1**) elicited a rebound burst following hyperpolarization, while P2 did not (**B2**). Interchanging either the *I_h_* conductance (**C**) or the *Ca_T_* conductance (**D**) between P1 and P2, leaving all other parameters the same, reduced the rebound response in P1, and increased it in P2. Interchanging both the *I_h_* and *Ca_T_* conductances between P1 and P2, also interchanged their bursting abilities completely (**E**). The scale bar applies to all panels. When conductance values were changed, the resting potential was kept at the original level by small compensatory current injections.

In order to elicit bursts, a preceding hyperpolarization of the membrane potential is often required [36], [53]. However, the somatic current injections used *in vitro* to study this effect do not occur *in vivo*. For example, the -150 pA current injections used in our experiments gave rise to a membrane potential much more hyperpolarized than the typical GABAergic reversal potential. We therefore simulated a more realistic, synaptic GABAergic input, to investigate whether our model could elicit rebound bursts under more realistic conditions. We found that 50 synapses of moderate strength (maximum conductances of 1 nS), activated with 10 ms intervals over a time period of 300 ms, reproduced the essential findings from the current-clamp experiments. When held at normal resting potentials, no rebound burst was elicited in either of the parameterizations (Figure 7A). However, when held at -57 mV, the series of synaptic activations provoked a rebound burst in P1, but not in P2 (Figure 7B).

Rebound bursts in various neurons are normally mediated by *Ca_T_* and/or *I_h_* channels [39], [64], [84]–[86]. We used the simulation setup with synaptic input to explore the contributions from these two ion channels to the rebound bursts in INs. In the original parameterizations (Table 1), P1 had higher values than P2 for both *g_CaT_* and *g_h_*, which explains why P1 and not P2 elicited rebound bursts. When *g_h_* was interchanged between P1 and P2, leaving all other parameters as in Table 1, the rebound burst in P1 was reduced to include only a single AP, while the (sub-threshold) rebound response in P2 became more pronounced (Figure 7C). Similar results were obtained when *g_CaT_* was interchanged between the two parameterizations: The rebound response in P1 fell below the AP firing threshold, while the (sub-threshold) rebound response in P2 became stronger (Figure 7D). Finally, if both parameters (*g_h_* and *g_CaT_*) were interchanged between P1 and P2, also the rebound responses were entirely interchanged: P1 showed a small sub-threshold rebound response, while P2 elicited a pronounced rebound burst, resembling that originally seen in P1 (Figure 7E). This suggests that *Ca_T_* and *I_h_* are of comparable importance for rebound bursts in mouse INs.

### Dependence on morphology

To investigate how sensitive our results are to the IN morphology, we ran test simulations using three additional IN morphologies with (a) a similar, (b) a smaller and (c) a larger total membrane area compared to the original morphology (o). We replaced the original morphology (o) with the new morphologies (a–c), but kept all other model parameters fixed at the values in Table 1. As the ion channel densities (i.e. conductances per µm^2^) were kept fixed, the new morphologies (a–c) corresponded to INs with (a) a similar, (b) a higher, and (c) lower input resistance compared to the original morphology (o).

The AP shape was not strongly affected by the morphology changes, except from small variations in the afterdepolarization (Figure 5A). The I/O curve in cases (o) and (a) nearly coincided (Figure 5B). As expected from the differences in total input resistance, morphology (b) gave rise to a I/O curve that was steeper and shifted in the negative direction along the current axis, whereas morphology (c) gave rise to an I/O curve that was flatter and shifted in the positive direction along the current axis compared to the cases (o) and (a) (Figure 5B). Although occurring at different current input levels, the characteristic responses of P1 and P2 did not change qualitatively with morphology. Depolarizing current injections of sufficient intensity gave rise to initial bursts followed by regular AP firing, and with the initial bursts being most pronounced for the parameterization P2. Furthermore, strong hyperpolarizing current injections (-150 pA in case of morphology (o), (a) or (b) and -200 pA in case of morphology (c)) were followed by rebound bursts when using parameter set P1 at a holding potential of -57 mV, but not when using the parameter set P2 at a holding potential of -58 mV. The essential firing patterns of P1 and P2 were thus preserved under changes of morphology, and the I/O curve was always steeper for parameterization P1 (Figure 5C).

## Discussion

We have developed a compartmental IN model which stands out from previous models in several ways: Firstly, the model was constrained by a broader range of I/O data than previous models of INs. It was able to exhibit a range of the response patterns characteristic for INs, including (i) initial sags, (ii) rebound bursts, (iii) tonic AP-firing, (iv) initial bursts/spike-time adaptation, and (v) periodic bursting. The mechanisms that we used to model these features have been described earlier in different works (e.g. [36], [63], [64], [83], [84], [86], [87]. By constraining the conductances of the different ion channels to I/O data from single neurons under eight different experimental conditions, including quantitative data on firing frequency vs. stimulus amplitude for depolarizing stimuli (I/O curves), we were able to make more specific predictions on the contributions of each mechanism. Secondly, we used a much more realistic morphology compared to earlier IN models that include active conductances, opening the way to future studies investigating how different dendritic regions (that generally receive input from different sources [78]) process local I/O operations. Thirdly, dendritic signal propagation also depends on active dendritic properties. As the dendrites of INs are both pre- and postsynaptic, dendritic ion channels will shape both incoming and outgoing signals, and will have an important impact on the signaling between INs and other neurons. Unlike previous IN models [33]–[36], our model includes a spatial distribution of ion channels over the somatodendritic membrane that is consistent with the available empirical data.

### Ion channels

The set of seven ionic conductances that we used to fit the experimental findings (Table 1) is the same as in the previous model by Zhu et al. [36], but with kinetics that were updated to account for recent findings for activation/inactivation kinetics and somatodendritic distributions, and with conductance values constrained by a broader range of I/O data. Although these seven channel types were successful in reproducing the observed spiking patterns, we cannot exclude the presence of additional mechanisms, either overlapping with an included conductance type in terms of their action on the firing properties, or with a minor or negligible effect on the somatic response observed in the current clamp experiments.

Several of the included ion channels have well documented roles in INs, including the role of *Ca_T_* in burst generation [36], [39], [44], the role of *I_h_* in generating initial sags [41] and the role of *Ca_L_* in increasing the intracellular calcium concentration [51], [53]. *I_CAN_* is involved in making the dendrites leakier in connection with cholinergic modulation [52]. Its influence on the somatic response pattern of INs is less clear, although a previous modeling study has suggested that *I_CAN_* may be involved in generating plateau potentials and prolong bursts [36]. Due to the high calcium sensitivity of this channel [36], [88], we found that even small depolarizations of the membrane gave rise to a tonically active *I_CAN_*. In our model, the main function of *I_CAN_* was to reduce the magnitude of the depolarizing current required for the neuron to reach AP firing threshold.

In comparison to somatic voltage recordings from rat INs [39], [41], our recordings from mouse INs show more pronounced initial sags for strong hyperpolarizing current injections (see Figure 2A, -150 pA stimuli). This suggests that the kinetics of *I_h_* may differ between INs in rats and mice, as is the case in CA1 pyramidal neurons [89].This we confirmed by measuring the voltage dependence of *I_h_* in three mouse INs (Figure 3). Simulations with *I_h_* kinetics based on our own measurements not only agreed better with the sag-shape in the mouse INs, but also predicted that the impact of *I_h_* on rebound burst generation was comparable to that of *Ca_T_* (Figure 6). This differs from the situation in rat INs, where rebound responses are mainly due to *Ca_T_*, with no measurable contribution from *I_h_* [39]. Conversely, in CA1 pyramidal neurons it has been shown that rebound spiking can be generated by *I_h_* alone [86]. However, a joint involvement of *Ca_T_* and *I_h_* in burst generation, were found to underlie intrinsic rhythmic bursting in subpopulations of TCs during inattentiveness in guinea pigs [64], [84]. Intrinsic rhythmic bursting was not observed in our neurons.

The presence of *Ca_L_*-conductances in IN dendrites [51], [53], [54] makes it likely that also inhibitory, calcium-dependent mechanisms (such as *I_AHP_*) are present. The role of *I_AHP_* in INs has not been previously documented. Our model predicted that the interplay between *Ca_L_* and *I_AHP_* conductances was sufficient for explaining the modulation of the I/O curves, although additional mechanisms could be involved. For example, a slowly activating potassium channel (*K_M_*) with a high threshold and no inactivation gave similar results to our *Ca_L_* and *I_AHP_* mechanism, but without any calcium dependence (simulations were made using *K_M_* kinetics taken from [90], results not shown). However, no clear functional role could be assigned to *K_M_* other than that covered by *I_AHP_*. We thus did not explore this issue further, since *I_AHP_* gave a better agreement than *K_M_* with the time course of the intra-spike membrane potential and, especially, with the afterhyperpolarization following the initial bursts in P2. However, the possibility that *K_M_* and *I_AHP_* have overlapping functions in regulating the spiking frequency cannot be excluded. This could be experimentally tested by blocking the respective channels.

We presented two parameterizations of the model (P1 and P2), which reproduced the electrophysiological properties of two different INs (IN1 and IN2). Rebound bursts as those generally observed only in a small subset of INs [39] were elicited by P1 but not P2. On the other hand, P2 elicited more pronounced initial bursts than P1 when exposed to depolarizing stimuli. P2 also had a less steep I/O curve and required weaker depolarization than P1 in order to reach AP-firing threshold. Our simulations showed that differences between P1 and P2 in terms of response properties and preferred input conditions arose from relative differences in specific conductances. Our model thus supports the idea that conductances values (i.e. channel density) in different subgroups of INs may be tuned in such a way as to optimize network operation under different input conditions. Under *in vivo* conditions, changes in input conditions (e.g. in synaptic input and/or shifts in membrane potential) may be mediated by mGlu5-receptor activation, GABAergic input from the reticular nucleus, or cholinergic modulation [4], [53], [54], [91], [92].

### Dendritic signal propagation

Data on the somatic voltage responses to somatic current injections do not uniquely determine the distribution of passive and active properties in the dendrites (see e.g. [48], [93]). Assumptions on the somato-dendritic distributions of *Ca_T_* and *Ca_L_* in our model were therefore based on calcium imaging data [50], [51]. This may be very useful in future studies, as the specific sub-cellular localization of different types of calcium channels may be particularly important for their specific functional role [43]. The assumed distributions of the remaining ion-channels were based on what we judged to be the most relevant literature to date. With the assumption that dendritic *Na* and *K_dr_* densities were 10% of those in the soma, simulations showed that APs got broader during propagation in the dendrites, whereas the amplitude did not get significantly attenuated (results not shown). This is, at least at a qualitative level, in good agreement with recent experiments using voltage-sensitive dye [70].

We cannot exclude the possibility that important aspects of dendritic signaling are not captured by our model at this stage. For instance, there is an uncertainty regarding the distribution of our dendritic *Ca_T_* channels. We based our *Ca_T_* distribution on electrophysiological data [50], which indicated that the *Ca_T_* density increases with distance to soma, while other, anatomy-based studies, have indicated a uniform distribution of dendritic *Ca_T_* channels [94]. In test simulations we showed that we could obtain essentially the same results as in Figure 4 and Figure 5 also with a uniform *Ca_T_* distribution, simply by rescaling the total *Ca_T_* conductances (results not shown). However, although it may not be crucial for an IN's response to somatic current injections, the *Ca_T_* distribution will likely influence aspects of dendritic signaling, such as the probability for dendritic GABA-release.

A related source of uncertainty concerns the presence of dendritic A-type potassium channels (*K_A_*), which, suggested by early studies, counteracted the *Ca_T_*-channels in the dendrites of INs, and suppressed bursting [38]. This mechanism has, however, not been observed consistently, and several experiments have reported bursting INs [36], [39], [40], [43]. For thalamic reticular neurons, the ability versus inability to burst was rather explained by a varying density of *Ca_T_*-channels [95], as also fits well with our findings. *K_A_* channels are often most densely present in distal dendrites and might have an influence on backpropagating action potentials [96]–[98]. However, recent experiments on INs found that attenuation of dendritic Ca-signals was relatively small [53]. As the importance of *K_A_* is unclear, these channels were not included in our model. Test simulations, using a moderate *K_A_* density (channel kinetics from [97]) in the dendrites, did not affect the somatic response to somatic stimuli significantly (results not shown). Such channels could therefore be readily added to the model if future experiments identify a clear functional role of *K_A_* in IN dendrites.

Parts of the distal dendrites of INs form so called triadic synapses with axons from retinal ganglion cells and dendrites from TC neurons [4], [99]. In these triads, the IN terminals are, at the same time, postsynaptic to retinal input, and presynaptic to TC neurons. The conditions for GABAergic release from IN dendrites are not fully known, but may depend on intracellular calcium levels which are elevated by (backpropagating) sodium spikes as well as signals evoked by local synapses [4], [53], [54], [99]. It is known that cholinergic modulation reduces the membrane resistance of INs, through the M2-receptor mediated activation of *I_h_*, *I_CAN_* and a linear, unspecified potassium current [52]. During sleep, when the cholinergic tone tends to be low, interneurons are likely to be electronically compact. INs may then provide long range inhibition, as synaptic input at one location in the dendritic tree then result in GABA release throughout the dendritic and axonal arbors. During awake states, when the cholinergic tone is high, distal dendritic regions may become electronically isolated from each other and from the soma. During these conditions, the triads may function as independent units, being excited by the presynaptic retinal afferents and then directly inhibiting only the postsynaptic dendrites of TC neurons [33], [52], [100], [101]. In our model, somatic action potentials successfully invaded distal dendritic region, and it is thus likely that our parameterizations (P1 and P2), correspond to conditions with a low cholinergic tone.

The present model includes dendritic sodium-, potassium-, and calcium channels, and at least some of the mechanisms that are affected by cholinergic modulators. It computes the time course of the intracellular calcium levels in each compartment, and contains essential mechanisms for addressing signaling in IN dendrites on a fine spatiotemporal scale. In a network model including the dLGN circuitry, this will be of paramount importance for simulating the interactions between TCs and INs, as well as inputs to these cells from retina, cortex, thalamic reticular nucleus and possibly modulatory input from the brain stem.

## References

[1] Sherman SM, Guillery RW (2001). Exploring the Thalamus.

[2] Hamos JE, Horn SCV, Raczkowski D, Uhlrich DJ, Sherman SM (1985). Synaptic connectivity of a local circuit neurone in lateral geniculate nucleus of the cat.. Nature.

[3] Norton TT, Godwin DW (1992). Inhibitory GABAergic control of visual signals at the lateral geniculate nucleus.. Prog Brain Res.

[4] Sherman SM (2004). Interneurons and triadic circuitry of the thalamus.. TRENDS Neurosci.

[5] Blitz DM, Regehr WG (2005). Timing and specificity of feed-forward inhibition within the LGN.. Neuron.

[6] Steriade M, McCormick DA, Sejnowski TJ (1993). Thalamocortical oscillations in the sleeping and aroused brain.. Science.

[7] Steriade M, Contreras D, Amzica F, Timofeev I (1996). Synchronization of fast (30–40 Hz) spontaneous oscillations in intrathalamic and thalamocortical networks.. J Neurosci.

[8] Lumer ED, Edelman GM, Tononi G (1997). Neural dynamics in a model of the thalamocortical system. I. Layers, loops and the emergence of fast synchronous rhythms.. Cereb Cortex.

[9] Traub RD, Contreras D, Cunningham MO, Murray H, LeBeau FEN (2005). Single-column thalamocortical network model exhibiting gamma oscillations, sleep spindles, and epileptogenic bursts.. J Neurophysiol.

[10] Hill S, Tononi G (2005). Modeling sleep and wakefulness in the thalamocortical system.. J Neurophysiol.

[11] Izhikevich EM, Edelman GM (2008). Large-scale model of mammalian thalamocortical systems.. Proc Natl Acad Sci USA.

[12] Wörgötter F, Koch C (1991). A detailed model of the primary visual pathway in the cat: comparison of afferent excitatory and intracortical inhibitory connection schemes for orientation selectivity.. J Neurosci.

[13] Köhn J, Wörgötter F (1996). Corticofugal feedback can reduce the visual latency of responses to antagonistic stimuli.. Biol Cybern.

[14] Wörgötter F, Nelle E, Li B, Funke K (1998). The influence of corticofugal feedback on the temporal structure of visual responses of cat thalamic relay cells.. J Physiol.

[15] Troyer TW, Krukowski AE, Priebe NJ, Miller KD (1998). Contrast-invariant orientation tuning in cat visual cortex: thalamocortical input tuning and correlation-based intracortical connectivity.. J Neurosci.

[16] Hillenbrand U, van Hemmen JL (2000). Spatiotemporal adaptation through corticothalamic loops: a hypothesis.. Vis Neurosci.

[17] Kirkland KL, Sillito AM, Jones HE, West DC, Gerstein GL (2000). Oscillations and long-lasting correlations in a model of the lateral geniculate nucleus and visual cortex.. J Neurophysiol.

[18] Hayot F, Tranchina D (2001). Modeling corticofugal feedback and the sensitivity of lateral geniculate neurons to orientation discontinuity.. Vis Neurosci.

[19] Einevoll GT, Plesser HE (2002). Linear mechanistic models for the dorsal lateral geniculate nucleus of cat probed using drifting-grating stimuli.. Network, Physics.

[20] Mayer J, Schuster HG, Claussen JC (2006). Role of inhibitory feedback for information processing in thalamocortical circuits.. Phys Rev E.

[21] Wielaard J, Sajda P (2007). Dependence of response properties on sparse connectivity in a spiking neuron model of the lateral geniculate nucleus.. J Neurophysiol.

[22] Steriade M (1997). Synchronized activities of coupled oscillators in the cerebral cortex and thalamus at different levels of vigilance.. Cereb Cortex.

[23] Sherman SM, Guillery RW (2002). The role of the thalamus in the flow of information to the cortex.. Philos Trans R Soc Lond B Biol Sci.

[24] Jones EG (2007). The Thalamus..

[25] Rose RM, Hindmarsh JL (1985). A model of a thalamic neuron.. Proc R Soc London B.

[26] Huguenard JR, McCormick DA (1992). Simulation of the currents involved in rhythmic oscillations in thalamic relay neurons.. J Neurophysiol.

[27] McCormick DA, Huguenard JR (1992). A model of the electrophysiological properties of thalamocortical relay neurons.. J Neurophysiol.

[28] Destexhe A, Babloyantz A, Sejnowski T J (1993). Ionic mechanisms for intrinsic slow oscillations in thalamic relay neurons.. Biophys J.

[29] Antal K, Emri Z, Tóth TI, Crunelli V (1997). Model of a thalamocortical neurone with dendritic voltage-gated ion channels.. Neuroreport.

[30] Destexhe A, Neubig M, Ulrich D, Huguenard J (1998). Dendritic low-threshold calcium currents in thalamic relay cells.. J Neurosci.

[31] Emri Z, Antal K, Crunelli V (2003). The impact of corticothalamic feedback on the output dynamics of a thalamocortical neurone model: The role of synapse location and metabotropic glutamate receptors.. Neuroscience.

[32] Rhodes PA, Llinás R (2005). A model of thalamocortical relay cells.. J Physiol.

[33] Bloomfield SA, Sherman SM (1989). Dendritic current flow in relay cells and interneurons of the cat's lateral geniculate nucleus.. Proc Natl Acad Sci USA.

[34] Briska AM, Uhlrich DJ, Lytton WW (2003). Computer model of passive signal integration based on whole-cell in vitro studies of rat lateral geniculate nucleus.. Eur J Neurosci.

[35] Perreault M-C, Raastad M (2006). Contribution of morphology and membrane resistance to integration of fast synaptic signals in two thalamic cell types.. J Physiol.

[36] Zhu JJ, Uhlrich DJ, Lytton WW (1999). Burst firing in identified rat geniculate interneurons.. Neuroscience.

[37] Kosmidis EK, Vibert J-F (2002). Feed-forward inhibition in the visual thalamus.. Neurocomputing.

[38] Pape HC, Budde T, Mager R, Kisvarday ZF (1994). Prevention of Ca2+-mediated action potentials in GABAergic local circuit neurones of rat thalamus by a transient K+ current.. J Physiol.

[39] Pape HC, McCormick DA (1995). Electrophysiological and pharmacological properties of interneurons in the cat dorsal lateral geniculate nucleus.. Neuroscience.

[40] Williams SR, Turner JP, Anderson CM, Crunelli V (1996). Electrophysiological and morphological properties of interneurones in the rat dorsal lateral geniculate nucleus in vitro.. J Physiol.

[41] Zhu JJ, Uhlrich DJ, Lytton WW (1999). Properties of a hyperpolarization-activated cation current in interneurons in the rat lateral geniculate nucleus.. Neuroscience.

[42] Zhu JJ, Lytton WW, Xue JT, Uhlrich DJ (1999). An intrinsic oscillation in interneurons of the rat lateral geniculate nucleus.. J Neurophysiol.

[43] Pape HC, Munsch T, Budde T (2004). Novel vistas of calcium-mediated signalling in the thalamus.. Pflugers Arch.

[44] Broicher T, Kanyshkova T, Landgraf P, Rankovic V, Meuth P (2007). Specific expression of low-voltage-activated calcium channel isoforms and splice variants in thalamic local circuit interneurons.. Mol Cell Neurosci.

[45] Gulledge AT, Kampa BM, Stuart GJ (2005). Synaptic integration in dendritic trees.. J Neurobiol.

[46] Mainen ZF, Sejnowski TJ (1996). Influence of dendritic structure on firing pattern in model neocortical neurons.. Nature.

[47] Kath WL (2005). Computational modeling of dendrites.. J Neurobiol.

[48] Zomorrodi R, Kröger H, Timofeev I (2008). Modeling thalamocortical cell: impact of ca channel distribution and cell geometry on firing pattern.. Front Comput Neurosci.

[49] Nusser Z (2009). Variability in the subcellular distribution of ion channels increases neuronal diversity.. Trends Neurosci.

[50] Munsch T, Budde T, Pape HC (1997). Voltage-activated intracellular calcium transients in thalamic relay cells and interneurons.. Neuroreport.

[51] Budde T, Munsch T, Pape HC (1998). Distribution of L-type calcium channels in rat thalamic neurones.. Eur J Neurosci.

[52] Zhu JJ, Heggelund P (2001). Muscarinic regulation of dendritic and axonal outputs of rat thalamic interneurons: a new cellular mechanism for uncoupling distal dendrites.. J Neurosci.

[53] Acuna-Goycolea C, Brenowitz SD, Regehr WG (2008). Active dendritic conductances dynamically regulate GABA release from thalamic interneurons.. Neuron.

[54] Antal M, Acuna-Goycolea C, Pressler RT, Blitz DM, Regehr WG (2010). Cholinergic activation of M2 receptors leads to context-dependent modulation of feedforward inhibition in the visual thalamus.. PLoS Biol.

[55] Halnes G, Augustinaite S, Heggelund P, Einevoll GT, Migliore M (2010). A compartmental model of an LGN interneuron.. Soc Neurosci Abstract.

[56] Tamamaki N, Yanagawa Y, Tomioka R, Miyazaki JI, Obata K (2003). Green fluorescent protein expression and colocalization with calretinin, parvalbumin, and somatostatin in the GAD67-GFP knock-in mouse.. J Comp Neurol.

[57] Hines M, Carnevale NT (1997). The NEURON simulation environment.. Neural Comp.

[58] Chitwood RA, Hubbard A, Jaffe DB (1999). Passive electrotonic properties of rat hippocampal CA3 interneurones.. J Physiol.

[59] Mainen ZF, Sejnowski T, Koch C, Segev I (1998). Modeling active dendritic processes in pyramidal neurons.. Methods in Neuronal Modeling: from Ions to Networks, 2nd edn.

[60] Hodgkin AL, Huxley AF (1952). A quantitative description of membrane current and its application to conduction and excitation in nerve.. J Physiol Lond.

[61] Hodgkin AL, Katz B (1949). The effect of sodium ions on the electrical activity of the giant axon of the squid.. J Physiol.

[62] Traub RD, Miles R (1991). Multiple modes of neuronal population activity emerge after modifying specific synapses in a model of the CA3 region of the hippocampus.. Ann N Y Acad Sci.

[63] Pospischil M, Toledo-Rodriguez M, Monier C, Piwkowska Z, Bal T (2008). Minimal Hodgkin-Huxley type models for different classes of cortical and thalamic neurons.. Biol Cybern.

[64] McCormick DA, Bal T (1997). Sleep and arousal: thalamocortical mechanisms.. Annu Rev Neurosci.

[65] Sah P (1996). Ca(2+)-activated K+ currents in neurones: types, physiological roles and modulation.. Trends Neurosci.

[66] Faber, ESL, Sah P (2003). Calcium-activated potassium channels: multiple contributions to neuronal function.. Neuroscientist.

[67] Destexhe A, Contreras D, Sejnowski TJ, Steriade M (1994). A model of spindle rhythmicity in the isolated thalamic reticular nucleus.. J Neurophysiol.

[68] Bal T, McCormick DA (1993). Mechanisms of oscillatory activity in guinea-pig nucleus reticularis thalami in vitro: a mammalian pacemaker.. J Physiol.

[69] Hughes SW, Cope DW, Blethyn KL, Crunelli V (2002). Cellular mechanisms of the slow (1 Hz) oscillation in thalamocortical neurons in vitro.. Neuron.

[70] Casale AE, McCormick DA (2010). Properties of dendritic action potential propagation in GABAergic interneurons of the thalamus and neocortex.. Soc Neurosci Abstract.

[71] Williams SR, Stuart GJ (2000). Action potential backpropagation and somato-dendritic distribution of ion channels in thalamocortical neurons.. J Neurosci.

[72] Sejnowski TJ (2009). Consequences of non-uniform active currents in dendrites.. Frontiers of neuroscience.

[73] Marrion NV, Tavalin SJ (1998). Selective activation of Ca2+-activated K+ channels by co-localized Ca2+ channels in hippocampal neurons.. Nature.

[74] Dreyfus FM, Tscherter A, Errington AC, Renger JJ, Shin H-S et al (2010). Selective T-type calcium channel block in thalamic neurons reveals channel redundancy and physiological impact of I(T)window.. J Neurosci.

[75] Traub RD (1982). Simulation of intrinsic bursting in CA3 hippocampal neurons.. Neuroscience.

[76] Wang XJ (1998). Calcium coding and adaptive temporal computation in cortical pyramidal neurons.. J Neurophysiol.

[77] De Schutter E, Smolen P, Koch C, Segev I (1998). Calcium dynamics in large neuronal models.. Methods in Neuronal Modelling – From Ions to Networks, 2 edition.

[78] Montero VM (1991). A quantitative study of synaptic contacts on interneurons and relay cells of the cat lateral geniculate nucleus.. Exp Brain Res.

[79] Zhu JJ, Lo FS (1999). Three GABA receptor-mediated postsynaptic potentials in interneurons in the rat lateral geniculate nucleus.. J Neurosci.

[80] Xiang Z, Huguenard JR, Prince DA (1998). GABAA receptor-mediated currents in interneurons and pyramidal cells of rat visual cortex.. J Physiol.

[81] Cruikshank SJ, Lewis TJ, Connors BW (2007). Synaptic basis for intense thalamocortical activation of feedforward inhibitory cells in neocortex.. Nat Neurosci.

[82] Budde T, Biella G, Munsch, T, Pape HC (1997). Lack of regulation by intracellular Ca2+ of the hyperpolarization-activated cation current in rat thalamic neurones.. J Physiol.

[83] Engel J, Schultens HA, Schild D (1999). Small conductance potassium channels cause an activity-dependent spike frequency adaptation and make the transfer function of neurons logarithmic.. Biophys J.

[84] McCormick DA, Pape HC (1990). Properties of a hyperpolarization-activated cation current and its role in rhythmic oscillation in thalamic relay neurones.. J Physiol.

[85] Russier M, Carlier E, Ankri N, Fronzaroli L, Debanne D (2003). A-, T-, and H-type currents shape intrinsic firing of developing rat abducens motoneurons.. J Physiol.

[86] Ascoli GA, Gasparini S, Medinilla V, Migliore M (2010). Local control of postinhibitory rebound spiking in CA1 pyramidal neuron dendrites.. J Neurosc.

[87] Destexhe A, Mainen ZF, Sejnowski TJ, Koch C, Segev I (1998). Kinetic models of synaptic transmission.. Methods in Neuronal Modeling, 2^nd^ Ed.

[88] Partridge LD, Swandulla D (1988). Calcium-activated non-specific cation channels.. Trends Neurosci.

[89] Routh BN, Johnston D, Harris K, Chitwood RA (2009). Anatomical and electrophysiological comparison of CA1 pyramidal neurons of the rat and mouse.. J Neurophysiol.

[90] Hemond P, Epstein D, Boley A, Migliore M, Ascoli GA (2008). Distinct classes of pyramidal cells exhibit mutually exclusive firing patterns in hippocampal area CA3b.. Hippocampus.

[91] McCormick DA, Pape HC (1998). Acetylcholine inhibits identified interneurons in the cat lateral geniculate nucleus.. Nature.

[92] Cucchiaro JB, Uhlrich DJ, Sherman SM (1991). Electron-microscopic analysis of synaptic input from the perigeniculate nucleus to the A-laminae of the lateral geniculate nucleus in cats.. J Comp Neurol.

[93] De Schutter E, Bower JM (1994). An active membrane model of the cerebellar Purkinje cell. I. Simulation of current clamps in slice.. J Neurophysiol.

[94] Parajuli LK, Fukazawa Y, Watanabe M, Shigemoto R (2010). Subcellular distribution of α1G subunit of T-type calcium channel in the mouse dorsal lateral geniculate nucleus.. J Comp Neurol.

[95] Lee S-H, Govindaiah G, Cox CL (2007). Heterogeneity of firing properties among rat thalamic reticular nucleus neurons.. J Physiol.

[96] Hoffman DA, Magee JC, Colbert CM, Johnston D (1997). K+ channel regulation of signal propagation in dendrites of hippocampal pyramidal neurons.. Nature.

[97] Migliore M, Hoffman DA, Magee JC, Johnston D (1999). Role of an A-type K+ conductance in the back-propagation of action potentials in the dendrites of hippocampal pyramidal neurons.. J Comput Neurosci.

[98] Jerng HH, Pfaffinger PJ, Covarrubias M (2004). Molecular physiology and modulation of somatodendritic A-type potassium channels.. Mol Cell Neurosci.

[99] Koch C (1985). Understanding the intrinsic circuitry of the cat's geniculate nucleus: electrical properties of the spine-triad arrangement.. Proc R Soc Lond B.

[100] Cox CL, Sherman SM (2000). Control of dendritic outputs of inhibitory interneurons in the lateral geniculate nucleus.. Neuron.

[101] Steriade M (2003). Presynaptic dendrites of thalamic local-circuit neurons and sculpting inhibition during activated states.. J Physiol.

